# Supplementary material for: Inferring visual space from ultra-fine extra-retinal knowledge of gaze position
Source: Nat Commun. 2023 Jan 17;14:269. doi: 10.1038/s41467-023-35834-4 (PMC9845343; doi:10.1038/s41467-023-35834-4)
Supplement: Supplementary file 1 — Supplementary Information [file 41467_2023_35834_MOESM1_ESM.pdf]

Supplementary Information: Inferring visual space from ultra-fine extra-retinal knowledge of gaze position

Zhetuo Zhao[1,2], Ehud Ahissar[3], Jonathan D. Victor[4], Michele Rucci[1,2]

[1]Department of Brain and Cognitive Sciences and [2]Center for Visual Science, University of Rochester, NY, USA. [3]Department of Brain Sciences, Weizmann Institute of Science, Rehovot, Israel. [4]Feil Family Brain and Mind Research Institute, Weill Cornell Medical College, New York, NY, USA

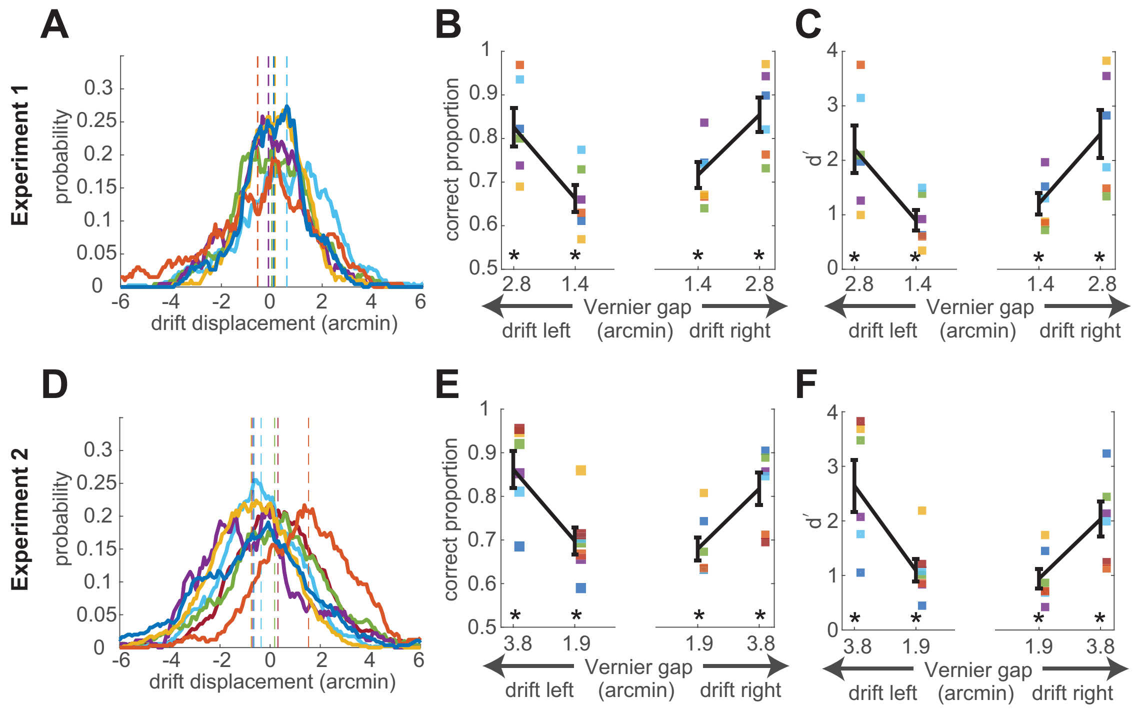

Fig. S1: **Performance as a function of ocular drift direction. (A-C)** Results from Experiment 1 (N=6 subjects). **(A)** Distributions of horizontal eye displacement in the 100 ms inter-stimulus interval of Experiment 1. Data from individual subjects are shown in separate curves. The vertical dashed lines mark the means of the distributions. Note that for all observers means are close to zero, *i.e.*, drift displacements were unbiased. **(B-C)** Performance in Experiment 1 measured as both proportion correct (B) and d' (C) for displacements in both directions. Black lines represent averages ± one SEM across subjects. Squares are data from individual subjects ($^\star p < 0.0035$ in B and < 0.0052 in C, two-tailed t-tests). Results with drifts in both directions were similar. **(D-F)** Similar analyses for the data from Experiment 2 (N=7 subjects) Graphic conventions are identical to the panels above, with black lines representing mean values ± SEM across subjects ($^\star p < 7.2 \times 10^{-4}$ in E and < 0.0027 in F, two-tailed t-tests). Source data are provided as a Source Data file.

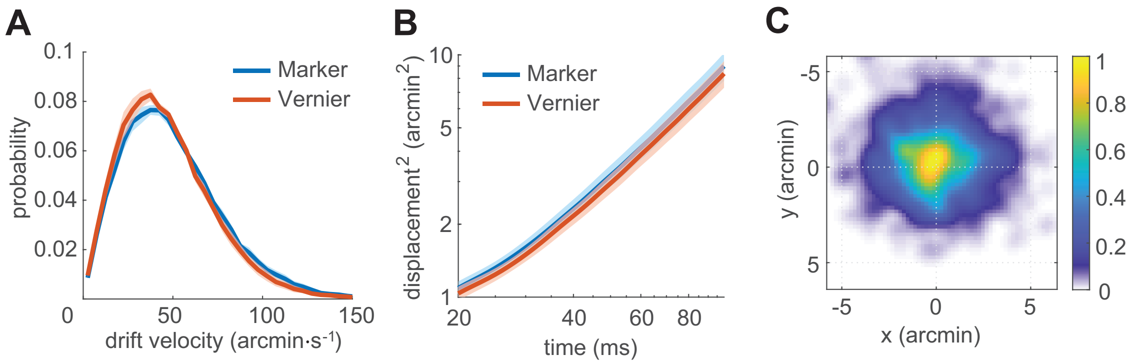

Fig. S2: **Ocular drift characteristics in Experiment 2.** (**A**) Average distribution of eye speed. (**B**) Squared displacement as a function of time. (**C**) 2D probability of overall drift displacement. Data represent averages across individuals and refer to the 100 ms ISI interval. Shaded regions represent ± one SEM. For comparison, the same measurements obtained while maintaining fixation on a 5′ dot (marker) are also shown in *A* and *B*. Source data are provided as a Source Data file.

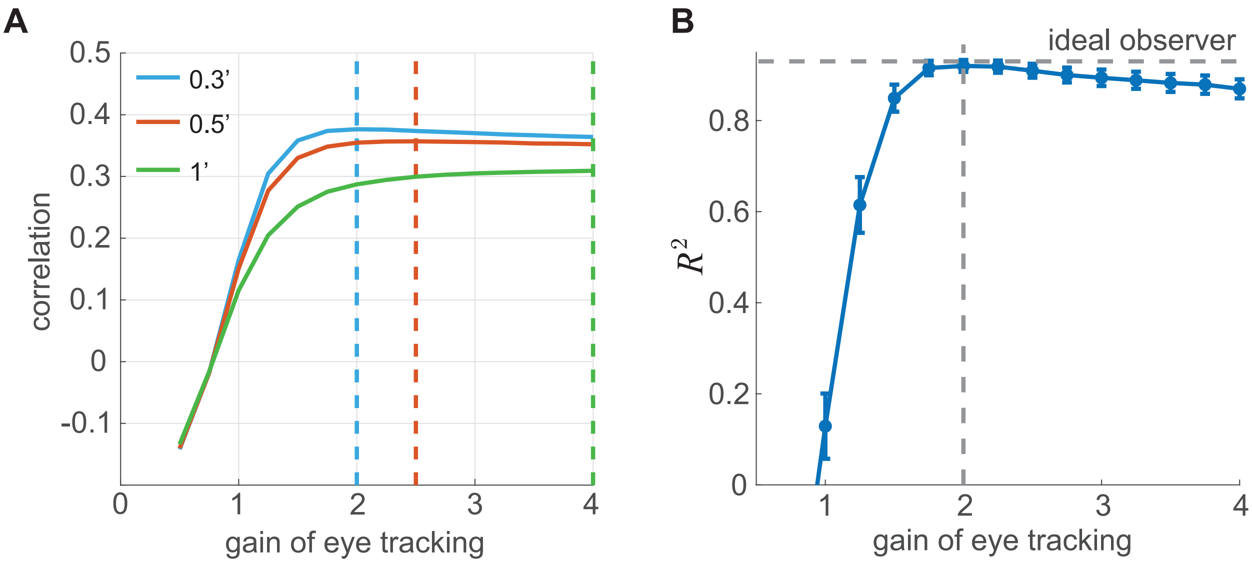

Fig. S3: **Consequences of over-estimating eye movements.** Results of Monte Carlo simulations that modeled the eye-tracker output as $\widehat{x} = \gamma x_e + \eta$, where x_e is the horizontal gaze displacement; γ represents the eye-tracker gain; and $\eta = N(0, \sigma)$ is a Gaussian noise term with zero mean and standard deviation σ_η. (**A**) Correlation between subject's responses and the resulting retinal misalignment $(X - \widehat{x})$ as a function of γ. The three curves represent results with different σ_η. The lower boundary for σ_η, as measured with a stationary artificial eye is 0.3. Note that the correlation never exceeds 0.4. Vertical dashed lines show the gains for which the curves reach their maximum. **(B)** Maximum variance in perceptual reports that could be explained by an ideal observer only using this retinal cue. For each γ, the perceptual uncertainty in the retinal measurement was estimated to maximize the R^2 as in Eq. 4. To account for subject's responses, the eye-tracker would need to overestimate the gaze displacement by approximately a factor of 2 (vertical line), which is unrealistic. The dashed horizontal line marks the variance accounted by the ideal observer in Figure 3, which assumes measurements of eye drifts to be veridical ($\gamma = 1$). For each gain, R^2 was evaluated over the $N = 124$ cue combinations of Fig. 3B. Errorbars represent $\pm$ one SEM from bootstrap. Source data are provided as a Source Data file.

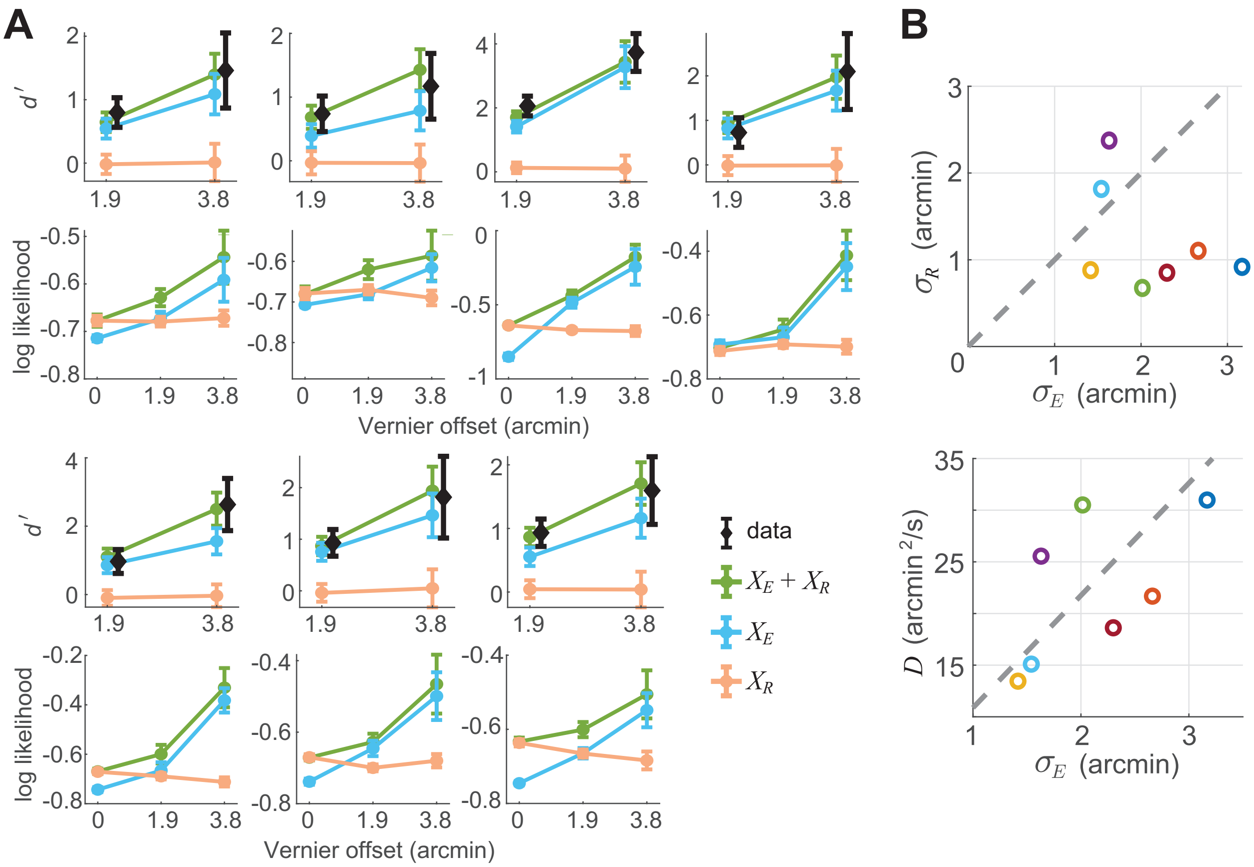

Fig. S4: **Model parameters and predictions for individual subjects.** **(A)** Three models are compared for each of the 7 subjects in Experiment 2: the full Bayesian model ($X_R + X_E$) and the two reduced models with a single cue (X_R or X_E). The performance of each model was evaluated by means of both predicted d′ (top row) and the mean log likelihood of all trials given the model (L in Eq. 4, see Methods. Bottom row). Errorbars are ± one SEM derived from bootstraps over an average of N=176 trials across subjects and gaps. **(B)** Model parameters fitted to empirical data in Experiment 2. Each data point corresponds to one subject: The s.d. of retinal noise σ_R and uncertainty in extraretinal displacement estimation σ_E in the top panel; The diffusion coefficient of ocular drift in the bottom panel. Source data are provided as a Source Data file.

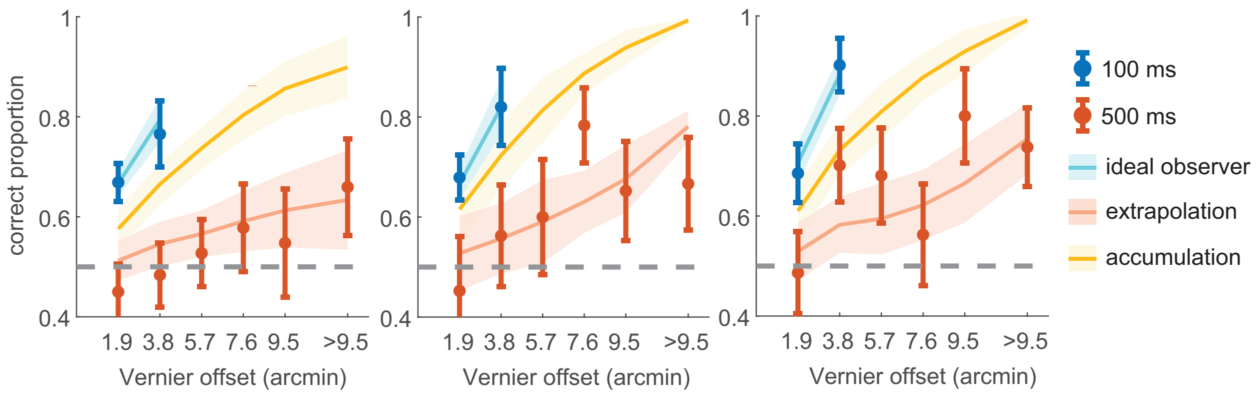

Fig. S5: **Model predictions for individual subjects in Experiment 3.** The graphical convention is the same as Fig. 4C. Error bars of the empirical data and the shaded region of model predictions are ± one SEM derived from bootstraps. Source data are provided as a Source Data file.

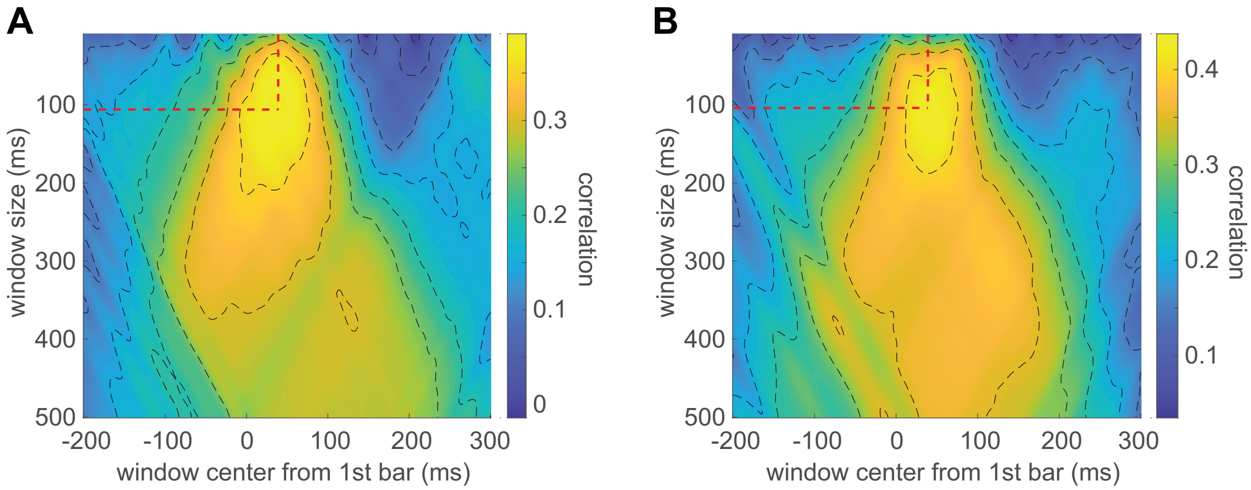

Fig. S6: **Extra-retinal strategies did not change over the course of training.** Correlation between gaze displacement and perceptual reports as a function of the position and duration of the window of observation, as in Fig. 4 (ISI=100). The two panels show averages obtained over the first (A) and last third (B) of the trials collected from each subject in Experiment 2.

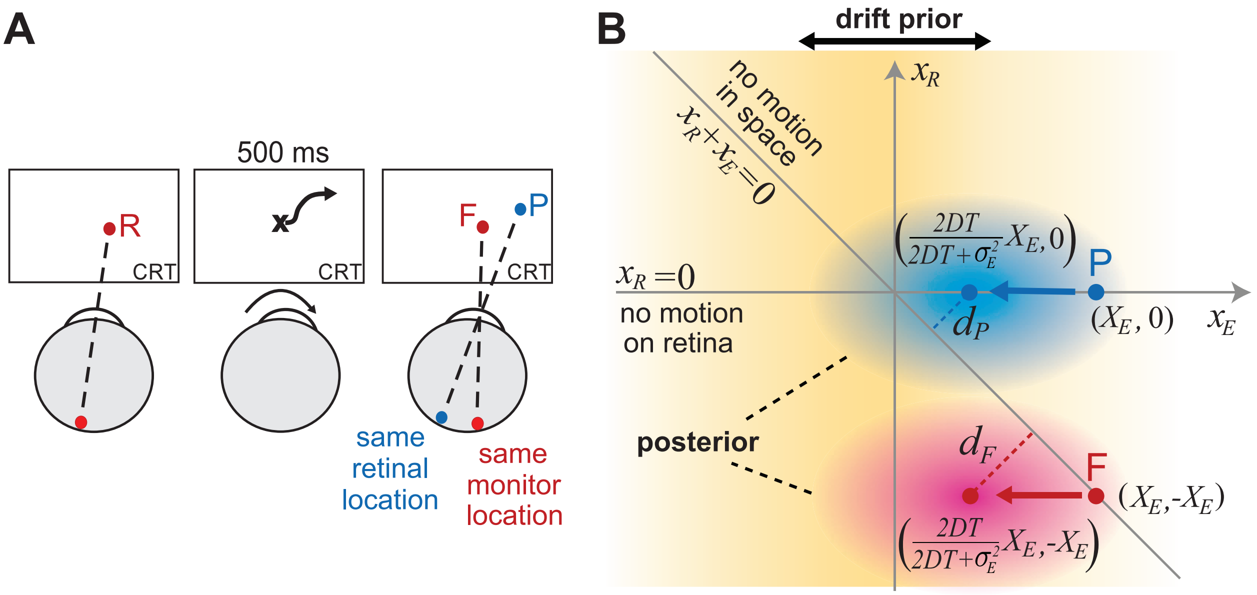

Fig. S7: **Predicted errors in spatial localization.** Our ideal observer model accounts for seemingly contradictory previous findings. (**A**) In a 2AFC task, subjects report the position of a previously displayed reference (R) by selecting between two probes, one at the same reference's location on the display (F) and one at its same position on the retina (P). The more the eye drifts in complete darkness, the less likely subjects are to correctly select F (see [29]). (**B**) The model predicts this paradoxical behavior as a consequence of the specific choice presented to the observer. The oculomotor prior weights identically both probes, causing both posterior distributions to shift towards smaller estimated displacements. The posterior distribution of the retinotopic probe P will be closer to the no-motion line (the line $X_R + X_E = 0$) if the motor uncertainty in measuring the displacement, σ_E, is larger than the variance of the prior ($\sigma_E^2 > 2DT$, where D is the drift diffusion constant and T the ISI). The data in Fig. 4 confirm that this will occur for sufficiently long ISI. Under these conditions, the model predicts that the retinotopic probe P will have higher probability to be mistaken for the reference than the spatiotopic probe F, despite having access to an extraretinal drift signal.
