# Supplementary material for: Inferring visual space from ultra-fine extra-retinal knowledge of gaze position
Source: Nat Commun. 2023 Jan 17;14:269. doi: 10.1038/s41467-023-35834-4 (PMC9845343; doi:10.1038/s41467-023-35834-4)
Supplement: Supplementary file 2 — Reporting Summary [file 41467_2023_35834_MOESM2_ESM.pdf]

nature portfolio

Corresponding author(s): Michele Rucci

Last updated by author(s): 2022/12/13

Reporting Summary

Nature Portfolio wishes to improve the reproducibility of the work that we publish. This form provides structure for consistency and transparency in reporting. For further information on Nature Portfolio policies, see our Editorial Policies and the Editorial Policy Checklist.

Statistics

For all statistical analyses, confirm that the following items are present in the figure legend, table legend, main text, or Methods section.

n/a	Confirmed	
☐	☒	The exact sample size (*n*) for each experimental group/condition, given as a discrete number and unit of measurement
☐	☒	A statement on whether measurements were taken from distinct samples or whether the same sample was measured repeatedly
☐	☒	The statistical test(s) used AND whether they are one- or two-sided<br>*Only common tests should be described solely by name; describe more complex techniques in the Methods section.*
☐	☒	A description of all covariates tested
☐	☒	A description of any assumptions or corrections, such as tests of normality and adjustment for multiple comparisons
☐	☒	A full description of the statistical parameters including central tendency (e.g. means) or other basic estimates (e.g. regression coefficient) AND variation (e.g. standard deviation) or associated estimates of uncertainty (e.g. confidence intervals)
☐	☒	For null hypothesis testing, the test statistic (e.g. *F*, *t*, *r*) with confidence intervals, effect sizes, degrees of freedom and *P* value noted<br>*Give P values as exact values whenever suitable.*
☐	☒	For Bayesian analysis, information on the choice of priors and Markov chain Monte Carlo settings
☒	☐	For hierarchical and complex designs, identification of the appropriate level for tests and full reporting of outcomes
☐	☒	Estimates of effect sizes (e.g. Cohen's *d*, Pearson's *r*), indicating how they were calculated

Our web collection on statistics for biologists contains articles on many of the points above.

Software and code

Policy information about availability of computer code

Data collection	Eye movement traces and subject responses were collected by means of EyeRIS, a custom hardware and software system for acquiring data and controlling stimulus display and timing (Santini et al, 2007). This system has been extensively tested and previously described (Rucci et al., 2007; Poletti et al., 2013).
Data analysis	Data were analyzed in Matlab R2021. Segmentation and characterization of recorded eye traces were performed based on instantaneous speed thresholds as summarized in the manuscript and previously described in the literature (Cherici et al., 2012; Poletti et al, 2013; Yu et al., 2018). A copy of the Matlab code is available at https://doi.org/10.5281/zenodo.7433824.

For manuscripts utilizing custom algorithms or software that are central to the research but not yet described in published literature, software must be made available to editors and reviewers. We strongly encourage code deposition in a community repository (e.g. GitHub). See the Nature Portfolio guidelines for submitting code & software for further information.

Data

Policy information about availability of data

All manuscripts must include a data availability statement. This statement should provide the following information, where applicable:

- Accession codes, unique identifiers, or web links for publicly available datasets
- A description of any restrictions on data availability
- For clinical datasets or third party data, please ensure that the statement adheres to our policy

Data are available from the Harvard Dataverse at https://doi.org/10.7910/DVN/FYKP574. Source data are provided with this paper

Human research participants

Policy information about studies involving human research participants and Sex and Gender in Research.

Reporting on sex and gender	Subjects consists of 5 males and 8 females;
Population characteristics	Age range: 20-35
Recruitment	Subjects were recruited by means of flyers and advertisements posted on the university campus and online. All volunteers were university students with normal, uncorrected vision and reflected the population distribution of students at the university. We have no reason to expect influences from self-selection bias or any other form of bias. The only noticeable consequence was that, since the majority of subjects were undergraduate students, the age span was limited.
Ethics oversight	The study protocol was approved by institutional review boards at Boston University and the University of Rochester

Note that full information on the approval of the study protocol must also be provided in the manuscript.

Field-specific reporting

Please select the one below that is the best fit for your research. If you are not sure, read the appropriate sections before making your selection.

☒ Life sciences ☐ Behavioural & social sciences ☐ Ecological, evolutionary & environmental sciences

For a reference copy of the document with all sections, see nature.com/documents/nr-reporting-summary-flat.pdf

Life sciences study design

All studies must disclose on these points even when the disclosure is negative.

Sample size	The subject sample size of subjects was chosen to ensure that significant effects generalize to a majority of the population when exhibited by each individual (see Anderson & Vingrys, 2001). Note that we are primarily interested in effects within individuals, not about population means. Thus, each individual datum in each subject is based on hundreds of trials.
Data exclusions	No subjects were excluded from this study. Criteria for selecting valid trials are described in Methods.
Replication	The same experimental procedure was replicated twice, Experiment 1 and 2, with different displays. Experimental findings were highly consistent across replications and also across individual observers, with each observer showing a significant effect. The experiments were broken up into multiple sessions (at least 4) over the course of several weeks to accumulate a sufficient number of trials for each participant. Data were consistent across sessions, and the summary results presented in the article are based on cumulative data from all sessions. The manipulation adopted in the study is conceptually simple and replication is straightforward, provided sufficiently accurate control of retinal stimulation.
Randomization	Participants were entered into experiments (N=6 for Experiment 1, N=7 for Experiment 2 100 ms ISI, and N=3 for Experiment 2 500 ms ISI) in the order in which they volunteered. Subjects were entered into more than one experiment if they consented. Within each experiment, all participants were in the same group, so the allocation method is not relevant. Trials were completed in random order.
Blinding	Investigators were de facto blinded to experimental conditions, as the stimulus configuration only depended on the eye movements performed by the subject and was unknown to the experimenter.

Reporting for specific materials, systems and methods

We require information from authors about some types of materials, experimental systems and methods used in many studies. Here, indicate whether each material, system or method listed is relevant to your study. If you are not sure if a list item applies to your research, read the appropriate section before selecting a response.

Materials & experimental systems

n/a	Involved in the study
☒	☐ Antibodies
☒	☐ Eukaryotic cell lines
☒	☐ Palaeontology and archaeology
☒	☐ Animals and other organisms
☒	☐ Clinical data
☒	☐ Dual use research of concern

Methods

n/a	Involved in the study
☒	☐ ChIP-seq
☒	☐ Flow cytometry
☒	☐ MRI-based neuroimaging
